# The Sympathetic Nervous System in Hypertensive Heart Failure with Preserved LVEF

**DOI:** 10.3390/jcm12206486

**Published:** 2023-10-12

**Authors:** Filippos Triposkiadis, Alexandros Briasoulis, Pantelis Sarafidis, Dimitrios Magouliotis, Thanos Athanasiou, Ioannis Paraskevaidis, John Skoularigis, Andrew Xanthopoulos

**Affiliations:** 1 School of Medicine, European University Cyprus, Nicosia 2404, Cyprus; 2 Department of Therapeutics, Heart Failure and Cardio-Oncology Clinic, National and Kapodistrian University of Athens, 115 27 Athens, Greece; alexbriasoulis@gmail.com; 3 Department of Nephrology, Hippokration Hospital, Aristotle University of Thessaloniki, 541 24 Thessaloniki, Greece; psarafidis11@yahoo.gr; 4 Unit of Quality Improvement, Department of Cardiothoracic Surgery, University of Thessaly, 411 10 Biopolis, Greece; dmagouliotis@gmail.com; 5 Department of Surgery and Cancer, Imperial College London, St Mary’s Hospital, London W2 1NY, UK; t.athanasiou@imperial.ac.uk; 6 6th Department of Cardiology, Hygeia Hospital, 151 23 Athens, Greece; iparas@otenet.gr; 7 Department of Cardiology, University Hospital of Larissa, 411 10 Larissa, Greece; iskoular@gmail.com

**Keywords:** hypertension, heart failure, sympathetic overactivity

## Abstract

The neurohormonal model of heart failure (HF) pathogenesis states that a reduction in cardiac output caused by cardiac injury results in sympathetic nervous system (SNS) activation, that is adaptive in the short-term and maladaptive in the long-term. This model has proved extremely valid and has been applied in HF with a reduced left ventricular (LV) ejection fraction (LVEF). In contrast, it has been undermined in HF with preserved LVEF (HFpEF), which is due to hypertension (HTN) in the vast majority of the cases. Erroneously, HTN, which is the leading cause of cardiovascular disease and premature death worldwide and is present in more than 90% of HF patients, is tightly linked with SNS overactivity. In this paper we provide a contemporary overview of the contribution of SNS overactivity to the development and progression of hypertensive HF (HHF) as well as the clinical implications resulting from therapeutic interventions modifying SNS activity. Throughout the manuscript the terms HHF with preserved LVEF and HfpEF will be used interchangeably, considering that the findings in most HFpEF studies are driven by HTN.

## 1. Introduction

According to the neurohormonal model of heart failure (HF) pathogenesis, sympathetic nervous system (SNS) overactivity is predominantly observed in HF with a reduced left ventricular (LV) ejection fraction (LVEF) [1]. In this regard, cardiac injury reduces cardiac output and the subsequent arterial underfilling is followed by activation of the SNS. In the short-term, SNS overactivity induces several changes in the heart, kidneys, and vasculature, which aim to maintain cardiovascular homeostasis. However, long-term SNS activation augments hemodynamic stress and exerts deleterious effects on the heart and the circulation [2].

The contribution of SNS overactivity in HF with preserved LVEF (HFpEF), which is due to hypertension (HTN) in the vast majority of the cases [3,4,5,6,7], has been undermined in this HF type [8,9]. Erroneously, HTN, which is the leading cause of cardiovascular disease and premature death worldwide [10], and according to the Framingham Heart Study is present in more than 90% of HF patients and increases the risk of incident HF in males and females two- and three-fold respectively [11], is tightly linked with SNS overactivity [12].

The purpose of this paper is to provide a contemporary overview of the contribution of SNS overactivity into the development and progression of hypertensive HF (HHF) [13], as well as the clinical implications resulting from therapeutic interventions modifying SNS activity. In this regard, following a brief discussion of the normal SNS cardiac control, we will summarize the contribution of SNS overactivity in HTN and HHF pathogenesis, and briefly describe the emerging medical and device modifications of SNS activity employed in HHF management. Throughout this manuscript the terms HHF with preserved LVEF and HFpEF will be used interchangeably as the findings in most HFpEF studies are driven by HTN [14,15,16].

## 2. Cardiac Sympathetic Control

The SNS is one the two anatomically distinct divisions of the autonomic nervous system (ANS), which controls cardiac function, the other being the parasympathetic nervous system (PNS) [17]. The ANS is generally organized based on the reflex arc, which has afferent and efferent limbs, and a central integration unit [18]. The afferent limb transmits information usually from peripheral baroreceptors and chemoreceptors to the central nervous system (CNS), whereas the efferent limb consists of preganglionic and postganglionic fibers and an autonomic ganglion. Simple reflexes are finalized within the involved organ, whereas complex reflexes are controlled by higher autonomic centers located mainly in the hypothalamus and brain stem. The paraventricular nucleus (PVN) of the hypothalamus is a distinctive key brain region involved in the control of cardiovascular, neuroendocrine, and other physiological functions [19]. The SNS has antagonistic albeit coordinated actions with the PNS. In this regard, the PVN (a central sympathetic nucleus) also projects directly to the nucleus tractus solitarious (NTS, a central parasympathetic nucleus), which receives the majority of cardiorespiratory afferents [20].

**Figure 1:**
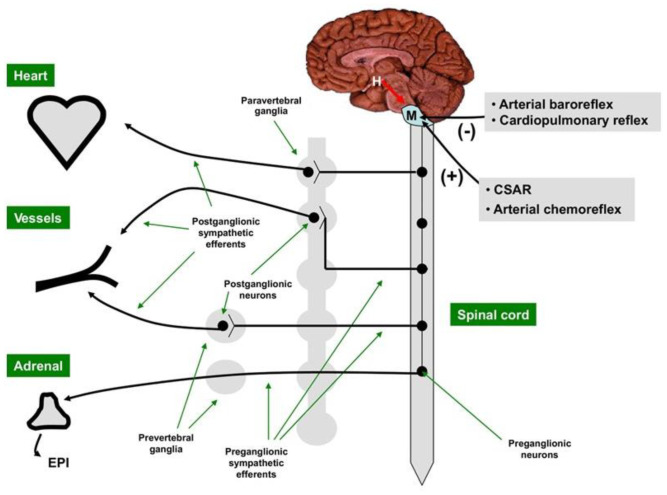
The central sympathetic nervous system [SNS] is located in the medulla and modulated by the hypothalamus. The motor outflow of the SNS is formed by two sets of neurons connected in tandem, the pre-ganglionic neurons originating in the brain stem or the spinal cord, and the post-ganglionic neurons located in the sympathetic ganglia. The main neurotransmitter of the SNS pre-ganglionic neurons is acetylcholine, while the main neurotransmitter of most SNS post-ganglionic neurons is norepinephrine. SNS activity is decreased (–) by the arterial baroreceptor reflex and the cardiopulmonary reflex and augmented (+) by the cardiac SNS afferent reflex (CSAR) and the arterial chemoreceptor reflex. H = hypothalamus; M = medulla. With permission from ref. [1].

The sympathetic nerves exit the medulla and travel down the spinal cord where they synapse with preganglionic fibers traveling to, and synapsing within, sympathetic ganglia. Postganglionic efferent fibers from the ganglia innervate the heart and vasculature. The adrenal medulla, which has features of both neural and endocrine systems, synthesizes the catecholamines epinephrine (EPI) and norepinephrine (NEPI) at a ratio of 4:1, which also serve as SNS neurotransmitters of the SNS postganglionic fibers [21] (Figure 1).

NEPI and EPI stimulate the cardiac β-adrenergic receptors (β-AR), which are members of the G protein-coupled receptors (GPCR). Three subtypes of β-AR have been identified, namely β1-AR, β2-AR, and β3-AR, with a fourth subtype (β4-AR) remaining controversial [22]. β1-AR is the dominant subtype in the normal heart, representing 75% to 80% of the total β-AR density, followed by β2-AR (15–18%) and β3-ARs (2–3%) [23]. β1-AR and β2-AR activation leads to positive inotropy, chronotropy, lusitropy, and dromotropy, whereas β3-AR seems to counterbalance cardiac adrenergic overactivity through a negative inotropic effect, involving the nitric oxide synthase (NOS) pathway [24], although the precise function of this AR subtype has not been delineated.

**Figure 2:**
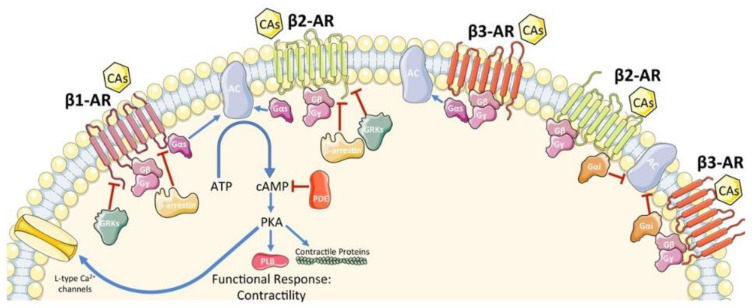
Schematic representation of β-AR signaling in cardiomyocytes. CAs, catecholamines; β-AR, β-adrenergic receptor; G protein subunits: Gα (Gαs or Gαi), Gβ, Gγ; GRK2, G protein-coupled receptor kinase 2; AC, adenylyl cyclase; ATP, adenosine tri-phosphate; cAMP, cyclic adenosine mono-phosphate; PDE, phosphodiesterase; PKA, protein kinase A. A blue arrow is used when a stimulatory mechanism is involved while a red bar-headed line is used for an inhibitory mechanism. With permission from ref. [25].

After binding the agonist, the β-AR undergoes a conformational change inducing the exchange in guanosine diphosphate (GDP) for guanosine triphosphate (GTP) and causing dissociation of the active Gα and Gβγ subunits. Although, all β-ARs are coupled with the stimulatory G protein (Gαs), β2-AR and β3-AR can also be coupled with the inhibitory G protein (Gαi) [25] (Figure 2). Activation of the Gαs subunit activates adenylyl cyclase (AC), whereas activation of Gai inhibits AC. AC activation converts adenosine triphosphate (ATP) into cyclic adenosine monophosphate (cAMP), which in turn activates protein kinase A (PKA) that phosphorylates effector molecules (e.g., phospholamban, L-type calcium channels, contractile proteins, and β-AR itself) leading to a functional response.

Prolonged stimulation of β1-AR induces cardiac hypertrophy and/or promotes cardiomyocyte apoptosis [26]. On the other hand, under stress conditions (e.g., hypoxia), β2-AR specific stimulation activates the Gαi and phosphoinositol 3-kinase dependent anti-apoptotic pathways, in contrast to β1-AR stimulation [27]. β-ARs can be deactivated through the actions of GPCR kinases (GRKs) [28], which recruit β-arrestins, uncoupling the receptor from the G proteins and promoting receptor internalization and down regulation by a clathrin mediated process. Further, downstream of β-ARs, β-arrestins can terminate signaling by recruiting phosphodiesterases and diacylglycerol kinase, which contribute to the breakdown of secondary messengers [25]. Finally, β-arrestins can initiate signaling cascades independent of G w2 protein activation, whereas PKA is also able to induce both homologous desensitization as well as β-AR agonist independent-heterologous desensitization by phosphorylation of the receptor [29].

Within the heart there are several intracardiac neurons forming the *intracardiac ganglia (ICG)*. Each ICG consists of 200 to 1000 intracardiac neurons, and groups of ICG with interconnecting nerves form the ganglionated plexi (GP), which function as “integration centers” modulating the interactions between the extrinsic and intrinsic cardiac ANS [30]. In the atria, GP accumulates in distinct locations on the chamber walls. Specifically, the right atrial GP controls sinus node firing, while the GP located at the junction of the inferior vena cava and left atrium (LA) controls the atrioventricular node (AVN) [31]. Another region that is richly innervated by the ANS and has a high density of GP is the pulmonary vein (PV)–LA junction in which adrenergic and cholinergic nerves are closely located. The ventricular GP are primarily located at the origins of several major cardiac blood vessels.

## 3. SNS Overactivity in Hypertension

SNS overactivity contributes to the increase in blood pressure with aging. At age 20, muscle sympathetic nerve activity (MSNA) of males and females are similar but subsequently diverge, reaching, in women, a minimum at age 30 [32]. After 30, the MSNA increases in both sexes and, compared with age 30, the MSNA burst frequency at age 70 is 57% higher in males and three-fold higher in females, corresponding to increases in systolic blood pressure from 1 to 12 mmHg, respectively. Further, there is compelling evidence that SNS overactivity contributes to HTN development [33,34]. Data from the Tecumseh Study have shown that young borderline hypertensives, especially those with a hyperkinetic circulation, have tachycardia at rest accompanied by elevated plasma NEPI [35]. Further, microneurographic studies have reported increased efferent sympathetic nerve traffic to the skeletal muscles in hypertensive patients [36]. Finally, studies using the NEPI spillover technique have shown an “actual” increase in NEPI secretion from the sympathetic nerve terminals in HTN, occurring in organs contributing to HTN development and progression, such as the kidney, or organs exposed to HTN complications, such as the heart and the brain [37,38].

The increased activity of SNS renal efferent nerves results in renal arteriolar vasoconstriction, reduced glomerular filtration rate (GFR), and stimulation of the renin-angiotensin aldosterone system (RAAS), ultimately leading to downstream salt and water retention [39]. Conversely, activation of renal afferents mediated by renal ischemia, hypoxia, or oxidative stress, facilitates increased stimulation of the hypothalamus as well as central SNS outflow to the juxtaglomerular apparatus, further increasing vascular resistance [40].

Arterial HTN in elderly patients derives from different pathophysiological mechanisms compared with middle aged or younger patients. The predominant increase in the systolic arterial pressure observed in the former patient population is associated with an increase in large artery stiffness (LAS) [41]. The increased LAS leads to the increased velocity of the incident wave and early return of the reflected wave and shifts pressure augmentation from diastole to systole. Consequently, systolic pressure and LV afterload rise. The SNS activity exhibits a positive association with LAS [42] as indicated by the fact that patients with NEPI-secreting pheochromocytoma have elevated adjusted (for age, blood pressure, heart rate, and fasting blood glucose) carotid-femoral pulse wave velocity (cf-PWV), a measure of arterial stiffness, which returns to normal one years after tumor resection, supporting a blood pressure-independent effect of chronic sympatho-adrenal overactivity on arterial stiffness [43].

## 4. SNS Overactivity in Hypertensive Heart Failure with Preserved LVEF

SNS activation has been considered a compensatory mechanism for the failing heart. Although this is true in the initial clinical phases of HF during which SNS overactivity plays a compensatory function aiming at the maintenance of an adequate cardiac output despite the presence of myocardial dysfunction, long-term SNS overactivity triggers a series of unfavorable remodeling processes, causing HF progression and the occurrence of major cardiovascular events [44,45].

The adverse cardiac effects of SNS overactivity in HF have been predominantly studied in HF with reduced LV ejection fraction (LVEF). In this setting, SNS overactivity results in an increase in catecholamine and a NEPI spillover from the cardiac sympathetic endings [16], leading to chronic β-AR hyperstimulation and maladaptive GRK2 upregulation (GPCR kinase 2) [46]. The increase in circulating catecholamines and upregulation in cardiac GRK2 promote β-AR down-regulation, cardiac hypertrophy, and myocyte apoptosis [47]. Further, GRK2 recruits β-arrestin, which then competes with G proteins for interaction with the β-AR and limits their activation [48].

In HF with a dilated LV, the SNS is activated chronically due to diverse neural derangements acting in concert [49]. Arterial underfilling due to the decrease in cardiac output unloads the arterial baroreceptors that restrain central sympathetic outflow. Further, the ventricular mechanoreceptors, which elicit reflex sympathoinhibition in response to inotropic forces or intracavitary pressures may be less abundant (e.g., prior infarction), or stimulated less (e.g., depressed contractile function or impaired ventricular relaxation) [50]. A significant minority of patients exhibit increased sympathetic outflow at rest due to sensitization of the peripheral chemoreceptor reflex [51]. Augmented sympathoexcitatory input from muscle metaboreceptors, from pulmonary arteries, and from renal afferent nerves may also be present under resting conditions and amplified further during exercise [52].

Several lines of evidence indicate that SNS overactivity is also present in HHF with preserved LVEF. Indeed, the SNS overdrive of essential HTN is potentiated when LV hypertrophy and dysfunction ensue as demonstrated by microneurography [53]. Similar were the findings from HFpEF studies, in which most of the patients exhibited a hypertensive phenotype [13,14,15] and diverse technics were used to evaluate SNS activity. In these studies, approximately 10% of HFpEF patients had elevated plasma renin activity (PRA), aldosterone, and NEPI, whereas 16% had elevated two and 41% had elevated one of the aforementioned factors, and during a 5-year follow-up, survival decreased with the number of elevated neurohormones [54]. Further, MSNA was progressively and significantly increased from controls to HF with preserved, midrange, and reduced LVEF [55], and cardiac imaging with iodine-123-metaiodobenzylguanidine (123I-MIBG), which is an analogue of NEPI and is useful for the estimation of cardiac SNS activity, proved a powerful prognosticator in patients with HF regardless of the LVEF [56,57]; in arterial and coronary sinus blood samples, those with preserved LVEF exhibited both cardiac (increased transcardiac NEPI gradient) and systemic (increased arterial NEPI) SNS activation [58]. Finally, muscle sympathetic discharge excessively increased during dynamic cycling in HFpEF patients [59]. Based on the aforementioned, it was proposed that excessive SNS activity during exercise in HFpEF elevates peripheral vascular resistance and blood pressure and concomitantly limits skeletal muscle blood flow, resulting in exercise intolerance [60].

## 5. Evaluation of SNS Activity in Heart Failure

SNS overactivity, which is associated with a poor prognosis and is a major treatment target, is present in the vast majority but not in all HF patients [54]. SNS evaluation, therefore, should be mandatory prior to the initiation of treatment in this patient population. There are several noninvasive tests for this purpose, including the analysis of the heart rate and blood pressure, measurement of a NEPI spillover, microneurography, and imaging of cardiac sympathetic nerve terminals [61].

The *resting heart rate*, which is regulated by the balance between the SNS and PNS, is an independent predictor of outcomes in HF. The lowest risk is observed at a resting heart rate of 50–60 bpm in the sinus rhythm [62,63]. The estimation of the parameters of heart rate variations using electrocardiographic (ECG) recordings (*heart rate variability, HRV*) provides indirect measures of the ANS activity in the time and the frequency domains [64]. HRV measures in the time domain include: SDNN (standard deviation of normal-to-normal RR interval) and RMSSD (root mean square of successive difference of RR interval), whereas in the frequency domain, the high-frequency power (HFP) reflects the PNS activity, and the low-frequency power (LFP) is modulated by both the PNS and SNS. A decreased heart rate variability is associated with an increased risk for incident HF [65] and adverse outcome in established HF [66].

The balance between the SNS and PNS is fine-tuned, by *cardiovascular reflexes*, including the arterial baroreflex, peripheral and central chemoreflexes, cardiopulmonary mechanoreflex, and pulmonary stretch receptor reflexes, which are abnormal in HF [67]. The arterial baroreflex is the most frequently evaluated cardiovascular reflex and when depressed is associated with adverse outcomes [68].

Cardiac ANS causes the release of NEPI at nerve terminals, which binds to adrenergic receptors and, for 80%, is reuptaken by the fiber endings. However, a small fraction of the released NEPI spills over into the circulation [69]. Regional NEPI spillover can be measured and serves as an indicator for SNS activity. Cardiac NEPI spillover in HF is much higher than that of other organs (e.g., kidneys or lungs). Cardiac NEPI spillover and renal NEPI spillover are major independent predictors of mortality in HF [70,71].

*Microneurography* is a noninvasive means for measuring the SNS activity by recording the postganglionic sympathetic nerves firing in skeletal muscle and skin. Sympathetic firing directed to skeletal muscle blood vessels is markedly increased in HF [72,73]. The increase is already present in mild HF, becoming greater in severe HF; it is independent from an HF cause and is potentiated by concomitant morbidities [74,75]. Microneurography has an independent predictive value on mortality in HF [76].

**Figure 3:**
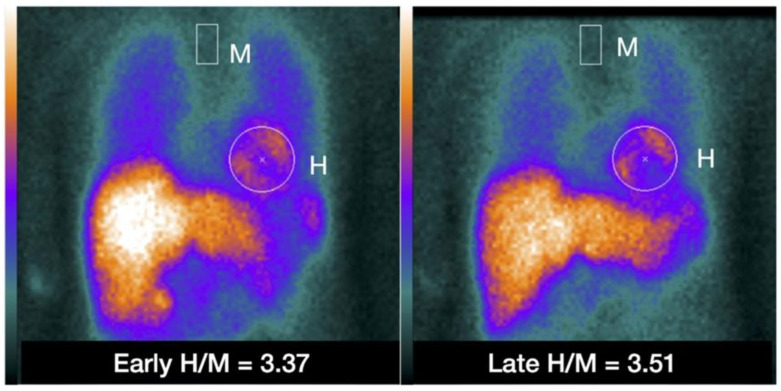
Example of placing a circular or elliptical region of interest (ROI) over the heart (H) and fixed rectangular mediastinal ROI placed on the upper part of the mediastinum (M) for calculating heart-to-mediastinum ratio (H/M). The same ROIs are placed on early and late images to calculate H/M and washout. The H/M outcomes are standardized to the ME-collimator condition. With permission from ref. [79].

Noninvasive *imaging techniques* using radiotracers, have been used to characterize the cardiac SNS with NEPI analogs: [123I] meta-iodobenzylguanidine [123I-MIBG] for single photon emission tomography [SPECT] imaging and [11C] meta-hydroxyephedrine [11C-HED] for positron emissions tomography [PET] imaging [77]. A reduced 123I-MIBG uptake, measured by the lower heart-to-mediastinum ratio (H/M), increased myocardial 123I-MIBG washout rate, and a reduced 11C-HED uptake during PET imaging, all represent cardiac sympathetic denervation [78,79] (Figure 3). Both low 123I-MIBG H/M and reduced 11C-HED uptake are markers of cardiac dysfunction and adverse outcomes in HF patients [57,80].

Some of the tests currently employed in the evaluation of SNS derangements in HF are not specific (e.g., heart rate) and other cumbersome and expensive (e.g., microneurography, SNS nuclear imaging) factors that hinder their widespread use. Further, interpretation of these tests is based on the understanding of cardiac sympathetic innervation and regulation, which is far from perfect. It is anticipated that technological improvements will contribute to the introduction of cardiac neurotransmission imaging in the every-day clinical praxis.

## 6. Therapeutic Implications

### 6.1. Medical Neuromodulation

**Figure 4:**
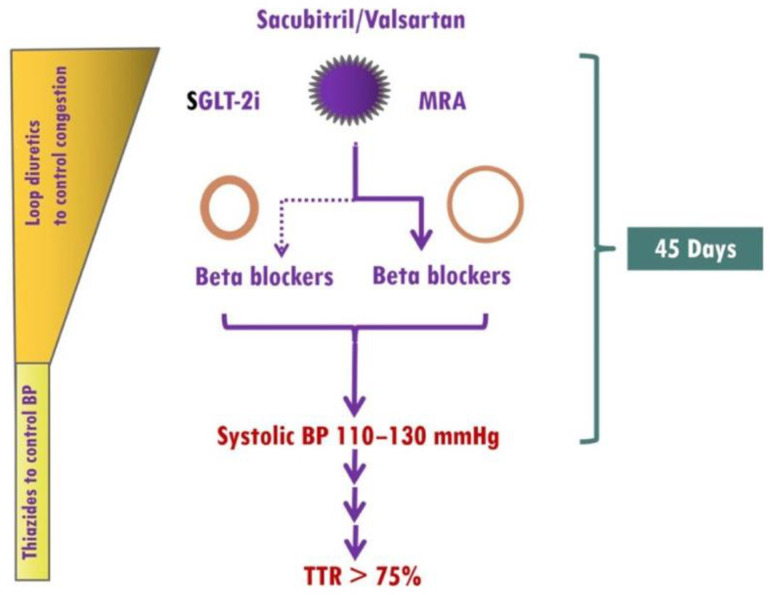
Implementation of medical treatment in hypertensive heart failure (HHF). The presence of elevated blood pressure (BP) in HHF as well as the renoprotective effects of finerenone, which rarely causes early hyperkalemia, allows ultra-fast up-titration of HF medications. Treatment should start with the simultaneous use of sacubitril/valsartan, sodium glucose cotransporter 2 inhibitors (SGLT-2i), and mineralocorticoid receptor antagonists (MRAs, preferably finerenone). In HHF patients with eccentric left ventricular hypertrophy (LVH), β-blockers (preferably vasodilatory) should be started from the beginning, whereas in HHF patients with concentric LVH, β-blockers should be considered in those with atrial fibrillation, coronary artery disease, or resistant hypertension. A target systolic BP of 110–130 mmHg should be achieved within 45 days, and thereafter, systolic BP should remain within the therapeutic target range most of the time. TTR, Time in Therapeutic Range. With permission from ref. [13].

Current drug treatment for HF, regardless of the cause and the LVEF, includes, in the absence of specific contraindications, treatment with (i) selected β-blockers, (ii) RAAS inhibitors (RAASi): angiotensin-converting enzyme inhibitors (ACEI), angiotensin receptor blockers (ARB), angiotensin receptor neprilysin inhibitors (ARNI), and mineralocorticoid receptor antagonists (MRA), and (iii) sodium-glucose cotransporter 2 inhibitors (SGLT-2i) [6,15,16,81,82] (Figure 4). Two common features of the aforementioned medications are the reductions of blood pressure and sympathoinhibition.

β-blockers are frequently used in HTN treatment. When treating HTN the different pathophysiology of HTN in the young (predominant SNS hyperactivity) and elderly patients (increased large artery stiffness, high aortic systolic pressure) should be considered with the use of non-vasodilating β-blockers (e.g., bisoprolol), preferred, as first-line, in young/middle aged hypertensive subjects and vasodilating β-blockers (e.g., carvedilol or nebivolol) in elderly hypertensives [83]. The downgrading of β-blockers by the European HTN guideline is not justified [84]. Regarding the effectiveness of β-blockers in HHF with preserved LVEF, a recent analysis of the Swedish HF registry (SwedeHF) demonstrated that β-blockers significantly reduced all-cause mortality and non-cardiovascular (CV) hospitalization in the atrial fibrillation (AF)-hypertensive cluster and lowered non-CV hospitalization in the obese-diabetic cluster [85]. Concerning the effect of β-blockers on cardiac SNS activity, the administration of β1-selective agents (e.g., bisoprolol) is associated with unaltered or increased cardiac NEPI spillover, whereas the administration of nonselective agents (e.g., carvedilol) reduces NEPI spillover, suggesting that in HF patients, a nonselective β-blockade may have favorable inhibitory effects on cardiac SNS activity [86,87].

RAASi have proved tremendously effective both in the management of HTN [6] and HF with preserved LVEF [88,89,90,91]. Chronic HF is associated with an ANS imbalance characterized by an augmented SNS activity and an attenuated PNS. This derangement has been attributed to a rise in systemic and cerebral angiotensin II signaling due to the elevated plasma angiotensin II in patients with chronic HF. The increase in angiotensin II signaling enhances the activity of the sympathetic nerves through actions on both the central and peripheral sites during chronic HF. Activation of angiotensin II signaling in different brain sites, such as the paraventricular nucleus (PVN), rostral ventrolateral medulla (RVLM), and area postrema (AP) may increase the release of NEPI [92]. Aldosterone-induced SNS excitation depends upon the angiotensin II type I receptor (AT1R)-induced mitogen activated protein kinase (MAPK) signaling in the brain through a nongenomic mechanism, likely via an aldosterone-induced transactivation of the AT1R [93].

ARNI are made up of two drugs, namely sacubitril, a neprilysin inhibitor, and the ARB valsartan. Neprilysin is a ubiquitous metallopeptidase with diverse substrates, including vasoactive peptides with vasodilating effects, such as natriuretic peptides (NPs), adrenomedullin, and bradykinin, as well as peptides with vasoconstrictor effects. The result of neprilysin inhibition is an increase in the circulating levels of NPs, which have several cardioprotective effects counteracting the deleterious effects of RAAS and SNS activation [94].

SGLT-2i have revolutionized HF treatment [3,95,96]. The observation that SGLT-2i decrease blood pressure in the absence of an increased heart rate suggests, indirectly, that these medications may reduce SNS activity [97]. In this regard, there is accumulating evidence that SGLT-2i leads to an attenuation of SNS activity, inhibits NEPI turnover in brown adipose tissue, and decreases tyrosine hydroxylase, the enzyme which catalyzes the rate limiting step in catecholamines synthesis [98,99]. There is also evidence to suggest that the attenuating effects of SGLT-2i on SNS activity may be secondary to a decrease in kidney stress, resulting in the inhibition of the afferent sympathetic activation to the kidneys [100].

Needless to say, adherence to medications is of the upmost importance and in this regard the use of single-pill combinations to control blood pressure may be effective [101,102,103].

### 6.2. Nonpharmacological Neuromodulation

Nonpharmacological neuromodulation includes several approaches, such as renal denervation, splanchnic nerve denervation, and cardiac contractility modulation.

**Figure 5:**
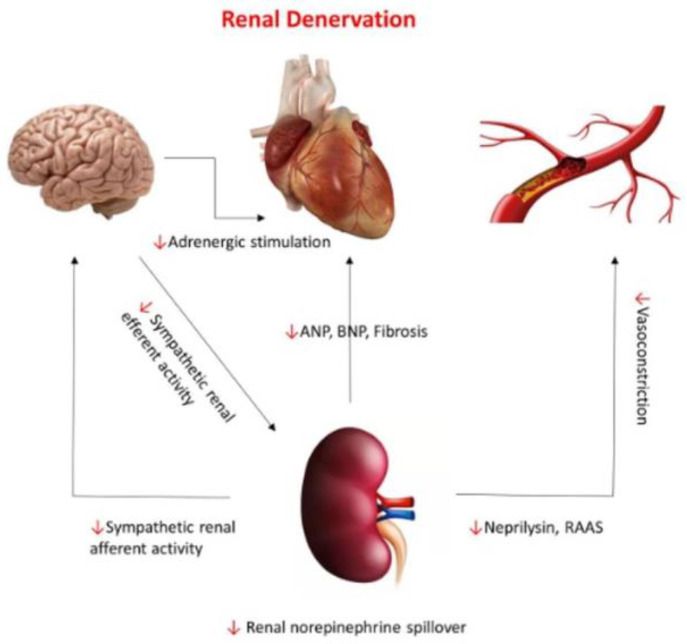
Neurohormonal pathways connecting the renal system to the central nervous system, cardiac, and vascular systems and the effect of renal denervation on those pathways (red arrows). With permission from ref. [105].

Considering the tight interactions between HHF with preserved LVEF, HTN, and renal sympathetic activity, renal denervation has been promoted as a rational therapeutic approach in this patient population [104,105] (Figure 5). As the results of preliminary studies have been encouraging, renal denervation may prove useful in the management of patients with HHF and preserved LVEF, providing optimization of patient selection and improvement in ablation strategies [106].

Volume recruitment from the splanchnic compartment is an important physiological response to stressors, such as physical activity and blood loss [107]. However, in the presence of HF, the blood translocation from this compartment into the systemic circulation leads to increased cardiac filling pressures, dyspnea, and limitation of functional capacity. As a result, targeting the greater splanchnic nerve (GSN) has emerged as a potential therapeutic strategy in HHF with preserved LVEF patients and the preliminary findings show that surgical resection of the right GSN may improve functional capacity and life quality in these patients by reducing cardiac filling pressure during exercise [108]. Likewise, the preliminary findings of the Endovascular Ablation of the Right Greater Splanchnic Nerve in Subjects Having HFpEF (Rebalance-HF) trial were encouraging [109].

Beyond SNS overactivity, PNS withdrawal has also been documented in HFpEF patients, and potentially contributes to disease progression and adverse outcomes [30]. Although vagus nerve stimulation (VNS) may prove beneficial in these patients it has the major drawbacks of requiring invasive implantation and having device-related complications [110]. An alternative, recently developed technique is the transcutaneous VNS (tVNS), performed by the noninvasive stimulation of the auricular branch of the vagus at the tragus level. A recent study employing low-level tVNS (20 Hz, 1 mA below discomfort threshold, for 1 h daily for 3 months) in HFpEF patients reported improved global longitudinal strain and tumor necrosis factor with no device-related side effects [111].

## 7. GAPS in Evidence and Future Directions

Although HTN is the most common risk factor for HFpEF, occurring in more than 90% of patients in some randomized control trials, it usually coexists with several closely related morbidities, such as type 2 diabetes mellitus, obesity, and coronary artery disease, which may also contribute both to SNS overactivity and HF development.

As previously mentioned, there is compelling evidence based on epidemiological data supporting the notion that HTN is the predominant driver both of SNS overactivity and HF development in this setting. However, the contribution of the other morbidities may not be negligible, necessitating the development of technics to assess the relative contribution of each morbidity to SNS overactivity and HF development. In this regard, application of advanced phenotyping with the use of high-resolution omics to elucidate genotype, endophenotype, and clinical phenotype levels in HHF will be necessary in the future. Another important issue, which has to be resolved, is the introduction of technics evaluating the SNS in everyday clinical practice, since SNS exerts a pivotal role in HF pathogenesis and progression. To date, all the technics proposed to assess the cardiac SNS are burdened by intrinsic limitations, hindering their widespread implementation in clinical practice. Easy to measure and accurate biomarkers, reflecting cardiac SNS activity, which will potentially support the risk stratification and guide the clinical management of the complex HHF syndrome, are urgently needed; myocardial GRK2 might be one of them as it is upregulated in HF patients, causing dysfunctional β-adrenergic receptor signaling and myocardial GRK2 levels correlating with levels found in peripheral lymphocytes in HF patients. The previous studies have demonstrated that lymphocyte GRK2 protein levels can independently predict prognosis in patients with HF [112]; cardiac GRK2 is a candidate treatment target in gene therapy in HF [113] and GRK2 regulates complex signaling pathways leading to fibrosis [114], which is highly prevalent in HF. Further evidence is required; however, to ascertain the effectiveness of GRK2 or other candidate SNS biomarkers in HF risk stratification and guidance of HHF sympathoinhibitory treatment in routine clinical practice.

## 8. Conclusions

SNS overactivity contributes to the development of both HTN and HHF regardless of the LEVF. Medications exhibiting sympathoinhibitory effects are currently the treatment cornerstones. Implementation of invasive neuromodulatory interventions necessitates detailed evaluation of SNS activity, which is currently expensive and cumbersome and confined to a limited number of research laboratories. In this context, a biomarker approach reflecting SNS activity, which appears essential, is currently lacking.

## Data Availability

Not applicable.
